# c-Jun-N-Terminal Kinase Signaling Is Involved in Cyclosporine-Induced Epithelial Phenotypic Changes

**DOI:** 10.1155/2012/348604

**Published:** 2011-10-18

**Authors:** Nicolas Pallet, Eric Thervet, Dany Anglicheau

**Affiliations:** ^1^Unité INSERM U775, Centre Universitaire des Saints-Pères, Université Paris Descartes, 75006 Paris, France; ^2^Service de Néphrologie, Hôpital Européen Georges Pompidou, 75015 Paris, France; ^3^Service de Transplantation Rénale, Hôpital Necker, Université Paris Descartes, 75006 Paris, France

## Abstract

Tubular epithelial cells play a central role in the pathogenesis of chronic nephropathies. Previous toxicogenomic studies have demonstrated that cyclosporine- (CsA-) induced epithelial phenotypic changes (EPCs) are reminiscent of an incomplete epithelial to mesenchymal transition (EMT) in a TGF-**β**-independent manner. Furthermore, we identified endoplasmic reticulum (ER) stress as a potential mechanism that may participate in the modulation of tubular cell plasticity during CsA exposure. Because c-jun-N-terminal kinase (JNK), which is activated during ER stress, is implicated in kidney fibrogenesis, we undertook the current study to identify the role of JNK signaling in EPCs induced by CsA. In primary cultures of human renal epithelial cells, CsA activates JNK signaling, and the treatment with a JNK inhibitor reduces the occurrence of cell shape changes, E-cadherin downregulation, cell migration, and Snail-1 expression. Our results suggest that CsA activates JNK signaling, which, in turn, may participate in the morphological alterations through the regulation of Snail-1 expression.

## 1. Introduction

The pathogenesis of cyclosporine (CsA) nephrotoxicity is not fully understood, and many mechanisms have been proposed [1, 2], such as the epithelial to mesenchymal transition (EMT), a phenomenon that has been implicated in the initiation and development of chronic nephropathies [3–6]. Under the pressure of numerous facilitating factors, tubular cells can lose their epithelial phenotype, express mesenchymal markers, acquire migratory and invasive potential, and secrete extracellular matrix. EMT promotes the generation of myofibroblasts, and growing evidence has implicated this process in diseased kidneys, leading to renal fibrosis [4, 7–9]. 

A better understanding of the role of tubular cells and EMT in the development of kidney fibrosis may lead to the development of specific biomarkers of early graft damage and to the characterization of novel therapeutic targets. Transcriptomic analysis of tubular cell response to CsA has shown that CsA induces EMT *in vitro* in a transforming growth factor *β*- (TGF-*β*-) dependent manner [10]. In a previous study, we have shown that CsA promotes TGF-*β*-independent epithelial phenotypic changes (EPCs). However, we have not observed *de novoα*-smooth muscle actin (*α*-SMA) expression, suggesting that CsA induces an incomplete EMT. In addition, we have identified the endoplasmic reticulum (ER) stress as a potential mechanism that may participate in the modulation of tubular cell plasticity in CsA-induced EPCs [11]. However, the precise mechanisms by which CsA promotes EPCs remain elusive.

The signaling pathways that are mediated by c-jun N terminal kinase (JNK), which is activated during ER stress following TRAF-2 recruitment and activation through Ire1 signaling [12], integrate epithelial cell plasticity, cell migratory potential, and the disruption of cell-cell contact during morphogenesis [13–15]. Importantly, JNK mediates kidney fibrogenesis in a mouse model of unilateral ureteral obstruction [16]. Notably, TGF-*β* and Rho GTPases signaling pathways involve JNK [14, 17]. 

We undertook the current study to test whether JNK signaling promotes tubular EPCs following CsA exposure. Here, we demonstrated that CsA activated JNK signaling in human renal epithelial cells and that JNK inhibition reduced the CsA-induced E-cadherin downregulation, cell migration, and Snail-1 expression. Our results suggest that JNK signaling is involved in CsA-induced tubular EPCs.

## 2. Materials and Methods

### 2.1. Material and Reagents

All chemicals, including CsA, were obtained from Sigma-Aldrich (Saint Quentin Fallavier, France). The cell culture medium and the other cell culture products were from Life Technologies (Cergy Pontoise, France).

### 2.2. Cell Culture

Human renal epithelial cells (HRECs) were cultured according to previously published methods [18, 19]. We used 6 *μ*M CsA as a working concentration because this concentration did not induce excessive toxicity and promoted EPCs [11]. Moreover, this concentration is similar to that observed in clinical practice where CsA blood concentrations may be measured up to 2 *μ*M.

### 2.3. RNA Extraction and Quantitative Real-Time Polymerase Chain Reaction

Total RNA was extracted using the RNeasy Mini Kit (Qiagen, Courtaboeuf, France) following the manufacturer's protocol. Transcripts expression levels were quantified using an ABI PRISM 7900 sequence detector system (Applied Biosystems, Foster City, CA, USA). Fold changes for each tested gene were normalized to Ribosomal Protein L13A (RPL13A). The relative expression levels among samples were calculated using the comparative delta Ct (threshold cycle number) method with vehicle-treated samples as references [20]. The mRNA levels were quantified in triplicate using the following primers: E-cadherin sense 5′-TACGCCTGGACTCCACCTA-3′ and antisense 5′-CCAGAAACGGAGGCCTGAT-3′; Snail-1 sense 5′-ACCCACACTGGCGAGAAGCC-3 and antisense 5′-TTGACATCTGAGTGGGTCTG-3; RPL13A sense 5′-CCTCGAGGAGAACAGGAAAGAGA-3′ and antisense R5′-GAGGACCTCTGTGTATTTGTCAA-3′.

### 2.4. Protein Extraction and Western Blot Analysis

Total protein lysates from HRECs were separated by sodium-dodecyl-sulfate polyacrylamide gel electrophoresis under denaturing conditions and transferred to PVDF membranes (GE Healthcare, Aulnay sous bois, France). E-cadherin, pJNK and JNK were detected using rabbit antibodies (No. 4065, N°4668, N°9252; dilutions at 1 : 1000 or 1 : 100 for pJNK; Cell Signaling Technologies, Saint Quentin en Yvelines, France), Snail-1 using a rabbit antibody (No. 17732; dilution at 1 : 500; Abcam, Paris, France) and actin using a rabbit antibody (N°2668, dilution at 1 : 5000; Sigma Aldrich, Saint Quentin Fallavier, France). The membranes were incubated with a horseradish peroxidase-conjugated polyclonal secondary antibody (Dako, Trappes, France) followed by ECL reagent (GE Healthcare).

### 2.5. Viability Studies

The relative number of living cells per well was determined based on mitochondrial integrity using the 3-(4,5-dimethylthiazol-2-yl)-5-(3-carboxymethoxyphenyl)-2-(4-sul-fophenyl)-2H-tetrazolium (MTS) assay (Promega, Charbonnieres, France) according to the manufacturer's instructions. 

### 2.6. Wound Healing

HRECs were grown in plastic coverslips to 75% confluence and were exposed to 6 *μ*M CsA for 48 hours. A straight and uniform scratch was made through the monolayer with a 200 *μ*L plastic pipette tip. The wounded areas were marked and photographed at different timepoints.

### 2.7. Statistical Analysis

All data were expressed as means ± SEM of three different experiments. Biological and histological data were compared using nonparametric tests. We used the Mann-Whitney test for comparisons between the two groups. Statistical analyses were performed using Prism 4 software (GraphPad software). *P *values that were of less than 0.05 were considered significant. 

## 3. Results

### 3.1. CsA Activates JNK Signaling

**Figure 1:**
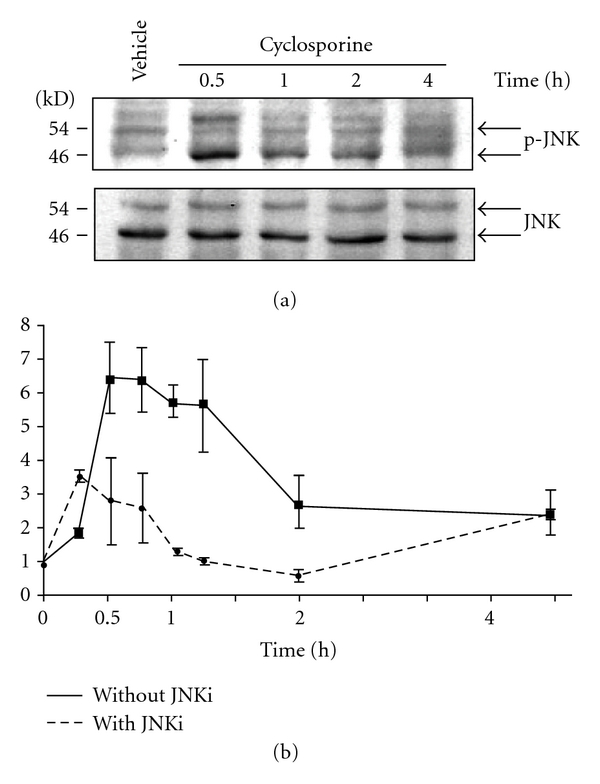
Cyclosporine (CsA) activates JNK signaling. (a) Representative western blot of JNK and p-JNK expression in primary cultures of HRECs exposed to the vehicle or 6 *μ*M CsA for various incubation times. (b) Real-time PCR analysis of EGR1 mRNA expression in cells that were exposed to 6 *μ*M CsA with or without the 2 *μ*M JNK inhibitor (L)-JNKI1.

We previously characterized CsA-triggered tubular EPCs and demonstrated that ER stress was implicated in these changes [11]. To understand how CsA induced EPCs, we focused on JNK signaling because JNK is activated during ER stress [21]. It has been shown to modulate cell plasticity and EMT, and is implicated in kidney fibrosis [13, 15, 16]. HRECs that were exposed to 6 *μ*M CsA displayed early JNK phosphorylation (Figure 1(a)). We inhibited JNK signaling using L-JNKi 1. The L-JNKi 1 represent the only potent inhibitors specific for JNK (JNK1, JNK2, and JNK3). Different from chemical inhibitors that directly affect kinase activity, for example, by competing with the ATP-binding site of the protein kinase, they rather inhibit the interaction between JNK and its substrate, resulting in a JNK K.O. phenotype [22]. JNK inhibition with (L)-JNKI1 attenuated the CsA-induced expression of early growth response 1 (EGR-1) mRNA, which is a JNK-dependent transcription factor [23]. These results suggest that CsA activates the JNK signaling pathway (Figure 1(b)). 

### 3.2. JNK Signaling Is Involved in CsA-Induced EPCs

**Figure 2:**
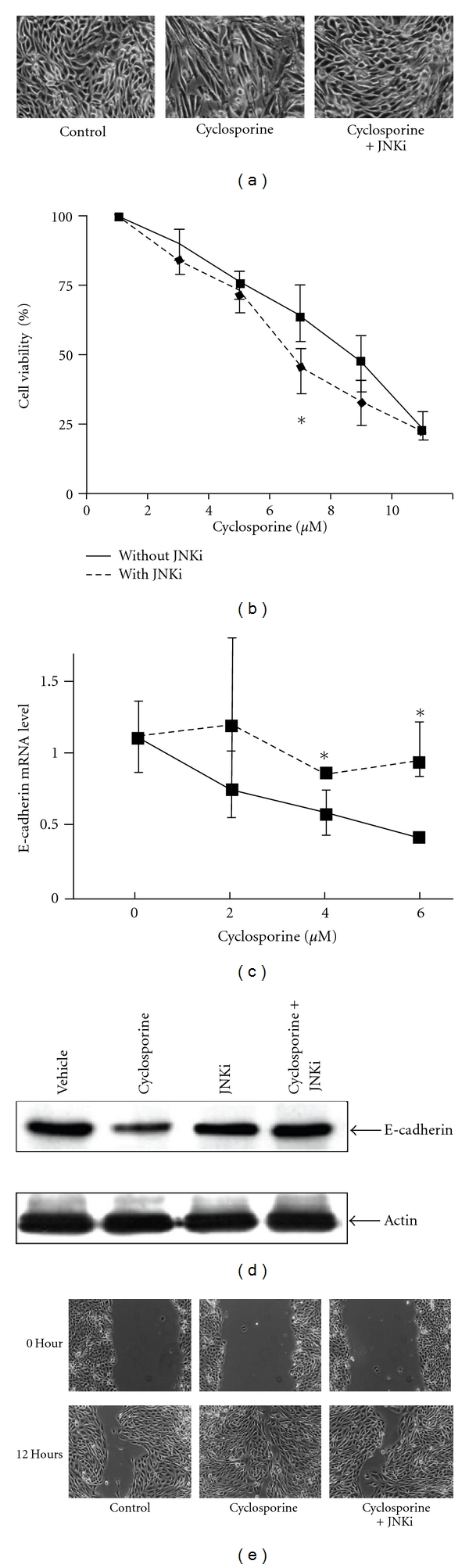
JNK signaling is involved in cyclosporine-induced EPCs. (a) Cellular morphology after 48 h exposure to 6 *μ*M cyclosporine with or without 2 *μ*M (L)-JNKI1 was determined using phase contrast microscopy. Bar, 4 *μ*m. (b). Cell viability was determined using the MTS assay after 72 h exposure to various concentrations of cyclosporine (CsA) with or without 2 *μ*M (L)-JNKI1; *P = 0.04, *n* = 4. (c) Real-time PCR analysis of E-cadherin mRNA expression after 48 h exposure to various concentrations of CsA with or without 2 *μ*M (L)-JNKI1. (d) Representative western blot of E-cadherin in whole cell lysates after exposure to vehicle, 6 *μ*M CsA, 2 *μ*M (L)-JNKI1, and 6 *μ*M CsA + 2 *μ*M (L)-JNKI1 for 48 hours. (e) Representative phase contrast micrographs of the initial wounding and 12 hours after wound healing (left). Bar, 50 *μ*m.

CsA-induced EPCs are characterized by an elongated shape, the development of lamellipodia, a large degree of cell detachment, and the downregulation of the epithelial marker E-cadherin (Figure 2(a) and [24]). To test whether JNK is implicated in these phenotypic changes, we treated HRECs with CsA in the presence or absence of the specific JNK inhibitor (L)-JNKI1. (L)-JNKI1 dramatically reduced the CsA-induced EPCs (Figure 2(a)), increased cell viability (Figure 2(b)), and suppressed CsA-induced E-cadherin downregulation at the mRNA (Figure 2(c)) and protein levels (Figure 2(d)). In addition, JNK inhibition reduced CsA-induced migratory properties of HRECs (Figure 2(e)). Together, these results suggest that the activation of JNK signaling by CsA regulates the phenotypic changes of HRECs.

### 3.3. CsA-Induced Snail-1 Expression Is JNK Dependent

**Figure 3:**
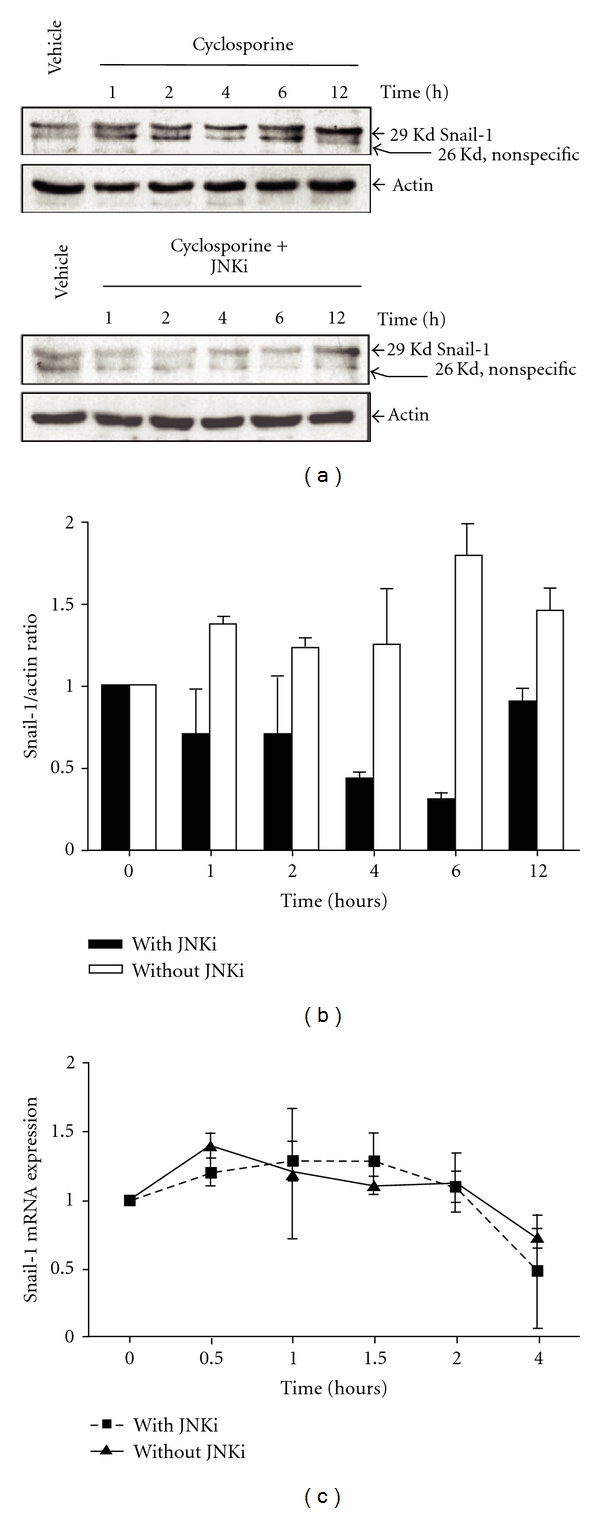
Cyclosporine induces *Snail-1 *expression in a JNK-dependent manner. (a) Representative western blot of Snail-1 expression in tubular cells during exposure for various incubation times with 6 *μ*M CsA or 6 *μ*M CsA + (L)-JNKI1. (b) Densitometric analysis of Snail-1 expression in three independent experiments. *P < 0.05 versus control. (c) Real-time PCR analysis of Snail-1 mRNA expression in tubular cells that were exposed for various incubation times with 6 *μ*M CsA with or without 2 *μ*M (L)-JNKI1.

Because JNK is implicated in CsA-induced EPCs, we tested whether JNK interacted with transcriptional factors that are implicated in EMT. Snail-1, which represses the expression of E-cadherin, is a central regulator of EMT. We demonstrated that CsA induced Snail-1 protein expression as early as one hour after treatment and that this upregulation was JNK dependent (Figures 3(a) and 3(b)). Interestingly, Snail-1 mRNA levels were not significantly altered by CsA treatment. This result suggests that CsA regulates Smail-1 mainly at the posttranscriptional level (Figure 3(b)). In addition, JNK inhibition did not affect the level of Snail-1 mRNA in CsA-treated cells, suggesting that the repression of CsA-induced Snail-1 expression is also posttranscriptionally determined (Figure 3(c)). These results suggest that CsA posttranscriptionally regulates *Snail-1* protein expression in a JNK-dependent manner. 

## 4. Discussion

In the current study, we showed that tubular cells that were exposed to CsA acquired phenotypic changes and that JNK signaling is implicated in this process. We focused on JNK activation because JNK is activated during ER stress and modulates cell plasticity at multiple levels. We found that in our model JNK was transiently activated during CsA exposure and that its inhibition attenuated CsA-induced EPC. The expression of the major E-cadherin repressor Snail-1 was increased after CsA treatment in a JNK-dependent manner. 

CsA induces a complete EMT *in vitro* through an autocrine and/or paracrine action of TGF-*β* [25]. TGF-*β* is the major inducer of EMT during chronic nephropathies, and CsA induces TGF-*β* synthesis and secretion *in vivo *[2]. Under our experimental conditions, CsA exposure did not induce TGF-*β* mRNA transcripts, the phosphorylation of Smad2, nor an alteration of Smad2/3 expression levels [24]. Therefore, TGF-*β* signaling was not a major contributor of the EPCs during CsA exposure in our model. 

 Our results indicate that the modulation of several characteristics of epithelial plasticity is reminiscent of an EMT and may be integrated by JNK activation during CsA exposure. JNK signaling is activated in various human kidney diseases including diabetic and hypertensive nephropathies [26]. In experimental models, JNK has been shown to mediate fibrogenesis in unilateral ureteral obstruction and antiglomerular basal membrane disease models (16, 26). Most importantly, JNK inhibition is beneficial in these models via its anti-inflammatory and antifibrotic properties. JNK is involved in fibrogenesis by mediating TGF-*β* signaling [17, 27]. Notably, TGF-*β*-independent activation of JNK is involved during EPCs and includes Rho GTPase signaling [17] and planar cell polarity [28]. Our results based on an *in vitro* model should be translated *in vivo* with caution. JNK signaling is believed to be a therapeutic target against fibrosis, and our results encourage further *in vivo* studies to test whether JNK inhibition limits the extension of fibrosis during chronic CsA-induced nephrotoxicity.

We demonstrated that the expression of transcriptional factor Snail-1 was regulated by JNK signaling. Snail-1 and Snail-2 (also known as Slug) belong to the Snail superfamily of zinc finger transcriptional repressors. They have emerged as key factors that regulate E-cadherin and the induction of EMT and are implicated in the pathophysiology of various human tumors [29]. In experimental kidney diseases, Snail-1 is overexpressed in renal tubular cells in the unilateral ureteral obstruction model, and its expression is posttranscriptionally stabilized by glycogen synthase kinase 3 *β* (GSK3*β*) [30]. Here, we demonstrated that the expression of Snail-1 at the protein level is dependent on JNK signaling during CsA treatment. JNK is known to regulate Snail-1 expression at the transcriptional level via the AP-1 transcription factor complex, which is a downstream target of JNK signaling [31]. However, the mechanisms by which JNK increases Snail-1 protein expression remain to be established.

In conclusion, our results demonstrate that CsA activates JNK signaling in tubular cells, leading to epithelial phenotypic changes. The result that JNK inhibition reverses this phenotype implicates JNK as a potential therapeutic target for the development of novel nephroprotective strategies.
